# Burden-dependent associations of serum sTREM2 with white matter injury and cognitive heterogeneity in cerebral small vessel disease

**DOI:** 10.3389/fnagi.2026.1937413

**Published:** 2026-09-21

**Authors:** Qiuju Gong, Xianfeng Yu, Shujian Chen, Ke Wan, Zhiqiang Wang, Bo Tian, Xiaoqun Zhu, Xia Zhou, Zhongwu Sun

**Affiliations:** 1 Department of Neurology, The First Affiliated Hospital of Anhui Medical University, Hefei, China; 2 Department of Clinical Pharmacology, The Second Hospital of Anhui Medical University, Hefei, China; 3 Chobanian & Avedisian School of Medicine, Boston University, Boston, MA, United States

**Keywords:** cerebral small vessel disease, cognitive impairment, diffusion MRI, microglia, neuroinflammation, soluble TREM2, white matter hyperintensities

## Abstract

**Objective:**

Cognitive outcomes vary among individuals with cerebral small vessel disease (CSVD), and conventional imaging markers may not fully explain this heterogeneity. Soluble triggering receptor expressed on myeloid cells 2 (sTREM2) is involved in TREM2-related myeloid responses, but the clinical significance of circulating sTREM2 in CSVD remains uncertain. We investigated the associations of serum sTREM2 with CSVD burden, white matter injury, and cognitive performance across different disease burdens.

**Methods:**

Serum sTREM2 levels were measured in 171 participants, including healthy controls and individuals with low- or high-burden CSVD. Associations between serum sTREM2 and total CSVD burden, individual imaging markers, white matter hyperintensity (WMH) volume, diffusion MRI measures, and cognitive performance were examined using multivariable regression models, tract-based spatial statistics, and burden-stratified analyses.

**Results:**

Serum sTREM2 levels increased across the CSVD burden spectrum and were associated with WMH volume, whereas no significant associations were observed with lacunes, cerebral microbleeds, or enlarged perivascular spaces. Higher serum sTREM2 was associated with widespread increases in mean diffusivity across major white matter tracts, while fractional anisotropy abnormalities were limited. The association between serum sTREM2 and Montreal Cognitive Assessment (MoCA) performance differed according to CSVD burden (interaction β = 0.462, HC3 *P* = 0.027), with a positive burden-stratified association observed in high-burden CSVD. Interpretation: Serum sTREM2 exhibited a white matter–predominant association pattern across the CSVD spectrum, and its relationship with cognitive performance varied according to disease burden. These findings suggest that circulating sTREM2 may provide additional information regarding biological heterogeneity in CSVD, although its cellular origin and longitudinal significance require further investigation.

## Introduction

Cerebral small vessel disease (CSVD) is a common late-life neurovascular disorder and a major contributor to stroke, cognitive impairment, and functional decline in older adults. Its principal neuroimaging manifestations include white matter hyperintensities (WMH), lacunes, cerebral microbleeds (CMBs), and enlarged perivascular spaces (PVS) (Duering et al., 2023; Wardlaw et al., 2019). These markers often coexist and are increasingly considered to represent a cumulative, brain-wide small vessel disease burden rather than isolated focal lesions (Debette and Markus, 2010; Staals et al., 2014, 2015). Among these imaging features, WMH and diffusion MRI-derived white matter microstructural abnormalities are closely related to cognitive impairment. However, cognitive performance varies substantially among individuals with comparable imaging burden, suggesting that structural lesion load alone does not fully explain clinical heterogeneity in CSVD.

Inflammation and innate immune activation have been increasingly implicated in the pathophysiology of CSVD (O’Donovan et al., 2021; Shahsavani et al., 2021). Chronic hypoperfusion, endothelial dysfunction, and blood–brain barrier disruption may promote a sustained inflammatory milieu within vulnerable white matter regions, involving both resident microglia and peripheral myeloid pathways (Shi and Wardlaw, 2016; Wardlaw et al., 2013, 2019). Circulating immune-related biomarkers are clinically attractive because they are accessible in peripheral blood, but their interpretation in CSVD remains challenging. Elevated biomarker levels may reflect greater tissue injury, biological responses to established damage, or both. Therefore, the clinical meaning of immune-related biomarkers may depend on the underlying burden of small vessel disease.

Triggering receptor expressed on myeloid cells 2 (TREM2) is an innate immune receptor expressed by microglia and peripheral myeloid cells and is involved in lipid sensing, phagocytosis, inflammatory regulation, and tissue injury responses (Foley, 2017; Suárez-Calvet et al., 2016). Its soluble form, soluble TREM2 (sTREM2), can be detected in cerebrospinal fluid and peripheral blood and has been proposed as a readout of TREM2-related myeloid activity (Bekris et al., 2018; Tsai et al., 2021). Evidence from neurodegenerative disease research suggests that the biological meaning of sTREM2 depends strongly on the underlying pathological context (Ewers et al., 2019; Ulland and Colonna, 2018). In cerebrovascular disease, plasma sTREM2 has been associated with WMH volume independently of amyloid and tau pathology (Tsai et al., 2021), supporting a potential link between circulating sTREM2 and small-vessel-related white matter injury.

However, it remains unclear whether circulating sTREM2 is associated with the overall burden of cerebral small vessel disease, whether its associations are more evident for diffuse white matter injury than for focal CSVD imaging markers, and whether its relationship with cognition varies according to CSVD burden. Therefore, we investigated the associations of serum sTREM2 with CSVD burden, white matter injury, and cognitive performance, and further examined whether the association between serum sTREM2 and global cognition differed across levels of CSVD burden. By integrating structural imaging, diffusion MRI, and cognitive assessments, we aimed to characterize the imaging and cognitive correlates of circulating sTREM2 across the CSVD spectrum.

## Materials and methods

### Participants

This was a single-center, cross-sectional observational study of participants recruited from the Department of Neurology, The First Affiliated Hospital of Anhui Medical University. The study protocol was approved by the Institutional Ethics Committee of Anhui Medical University (approval number: Quick-PJ 2023-13-62) and was conducted in accordance with the Declaration of Helsinki. Written informed consent was obtained from all participants before enrollment. Participants were eligible if they were aged 50–80 years, were able to complete the neuropsychological assessment, and had available brain MRI data. Patients with CSVD were further required to show one or more CSVD imaging markers on MRI. Potential control participants were recruited during the same study period from individuals who underwent brain MRI and neuropsychological assessment.

Exclusion criteria were: (i) acute infarction > 2 cm, cardioembolic stroke sources, intracerebral hemorrhage, malignancy, prior traumatic brain injury, or a history of neurosurgery; (ii) systemic infection or inflammatory disease, major organ dysfunction, or psychiatric or neurodegenerative disorders, including Alzheimer’s disease and Parkinson’s disease; (iii) use of medications that might affect sTREM2 levels or cognitive function; and (iv) CSVD secondary to defined causes, including genetic, infectious, autoimmune, neoplastic, toxic, radiation-induced, or metabolic etiologies.

### Serum sTREM2 measurement

Fasting venous blood ( ≥ 8 h) was collected, allowed to clot for 30 min at room temperature (25 °C), and centrifuged at 3,500 rpm for 8 min. Serum aliquots were stored at −80 °C until analysis. Serum sTREM2 levels were measured using an enzyme-linked immunosorbent assay (ELISA; Yiji Biotechnology, YiJi-14378) in a College of American Pathologists (CAP)–accredited laboratory. Calibration followed a five-parameter logistic (5PL) model, with detection and quantification limits of 1.0 and 2.25 pg/mL, respectively. All samples were analyzed in duplicate, and those with coefficient of variation (CV) > 10% were reanalyzed.

### Definition of CSVD markers and burden assessment

CSVD imaging markers were defined according to the Standards for Reporting Vascular Changes on Neuroimaging (STRIVE) and STRIVE-2 criteria (Duering et al., 2023; Wardlaw et al., 2013). WMH were rated on T2-weighted and fluid-attenuated inversion recovery images using the Fazekas scale (Fazekas et al., 1987). Lacunes, CMBs, and PVS in the basal ganglia were assessed on standard structural and susceptibility-sensitive MRI sequences (Potter et al., 2015). Detailed imaging definitions are provided in the Supplementary Methods.

The overall burden of CSVD was assessed using the composite MRI scoring system described by Staals et al (Staals et al., 2014). The total CSVD score ranges from 0 to 4, assigning one point for each feature present: (1) lacunes ( ≥ 1); (2) CMBs (any number detected on T2-weighted or SWI); (3) PVS in the basal ganglia ( > 10); (4) WMH, defined as confluent deep WMH (Fazekas grade 2–3) or periventricular WMH with irregular extension into deep white matter (Fazekas grade 3).

Participants were further stratified according to the total CSVD burden score. Healthy controls (HC) were defined as individuals with a total CSVD burden score of 0, no history of stroke or major neurological disease, and MoCA performance within the education-adjusted normal range. Low-burden CSVD (L-CSVD) and high-burden CSVD (H-CSVD) were defined as total CSVD scores of 1–2 and 3–4, respectively.

### Neuropsychological assessment

All cognitive assessments were administered by trained neurologists according to a standardized protocol. Global cognition was assessed using the Mini-Mental State Examination (MMSE) and Montreal Cognitive Assessment (MoCA). Language ability was assessed using the Boston Naming Test (BNT) (Williams et al., 1989), and verbal episodic memory was assessed using the Auditory Verbal Learning Test (AVLT). Executive control was assessed using the Stroop Color-Word Test (Part C), with completion time in the interference condition recorded; longer times indicated poorer executive control (Scarpina and Tagini, 2017), whereas attention and processing speed were assessed using the Trail Making Test–Part A (TMT-A) (Tombaugh, 2004). The Clinical Dementia Rating (CDR) global score was used to characterize overall clinical severity (Morris, 1993). Validated Chinese versions of the neuropsychological tests were used where applicable, and permission requirements were checked before use.

For the burden-dependent cognitive analyses, MoCA was selected as the primary measure of global cognition, whereas MMSE was examined as a complementary global cognitive measure. MoCA was selected because it assesses multiple cognitive abilities relevant to the multidimensional cognitive profile of CSVD and has been specifically validated in patients with cerebral small vessel disease. Previous work showed that both executive and non-executive cognitive abilities independently contributed to MoCA performance in this population (Wong et al., 2009). Given the recognized involvement of executive function and processing speed in CSVD, a composite measure reflecting executive function and processing speed was additionally examined as a secondary exploratory outcome. This composite was derived from Stroop-C and TMT-A (Salvadori et al., 2021). Completion times for both tests were log-transformed, z-standardized, directionally reversed, and averaged, with higher composite scores indicating better performance.

### MRI acquisition and image processing

MRI acquisition, preprocessing, and tract based spatial statistics (TBSS) analyses were performed in accordance with the best practice recommendations of the Organization for Human Brain Mapping (OHBM) Committee on Best Practices in Data Analysis and Sharing (Nichols et al., 2017).

Three dimensional structural MRI data were processed using the Computational Anatomy Toolbox (CAT12) (Gaser et al., 2024) in Statistical Parametric Mapping software (SPM12) (Malone et al., 2015). After bias-field correction, T1 images were segmented into gray matter (GM), white matter (WM), and CSF, and total intracranial volume (TIV) was calculated as GM + WM + CSF. WMH volume was quantified from T2-FLAIR images using the Lesion Segmentation Tool (LST v3.0.0; lesion prediction algorithm, LPA) (Heinen et al., 2019). Lesion probability maps were thresholded at 0.50 to generate binary lesion masks. The LST-derived WMH segmentation masks were independently visually inspected by two trained raters and manually corrected when necessary. Lacunes, CMBs, and PVS were independently assessed by the same raters according to established neuroimaging criteria. Both raters were blinded to participants’ clinical information, cognitive performance, serum biomarker measurements, and each other’s assessments. Inter-rater reliability was evaluated according to the type of imaging measure. Agreement in WMH volume, as a continuous measure, was assessed using a two-way mixed-effects intraclass correlation coefficient for absolute agreement based on single measurements (ICC[A,1]). For categorical ratings contributing to the total CSVD burden score, including the presence of lacunes, the presence of CMBs, and PVS > 10, inter-rater agreement was assessed using Cohen’s κ. Disagreements were resolved by consensus after joint review of the original images, with adjudication by a senior neuroradiologist when consensus could not be reached. Inter-rater reliability estimates are provided in Supplementary Table 1.

Diffusion MRI data were processed using the TBSS pipeline implemented in the FMRIB Software Library (FSL) (Jenkinson et al., 2012). Standard preprocessing steps included correction for eddy current induced distortions and head motion, followed by nonlinear registration of individual fractional anisotropy maps to the FMRIB58 FA standard space template. A mean FA image was then generated and thinned to create a white matter skeleton, thresholded at FA greater than 0.2 to restrict analyses to major white matter tracts. Voxel-wise permutation based general linear models with 5000 permutations were conducted using the randomize tool in FSL (Smith and Nichols, 2009) to examine the associations between serum sTREM2 levels and white matter microstructural integrity. Separate models included skeletonized FA or MD as dependent variables, z-standardized serum sTREM2 as the independent variable, and age, sex, and years of education as covariates. Multiple comparisons were corrected using Threshold-Free Cluster Enhancement (TFCE) (Smith and Nichols, 2009), with family-wise error (FWE)–corrected *p* < 0.05 considered statistically significant. Clusters smaller than 50 mm^3^ were excluded, and mean FA and MD values were extracted using *fslmeants* (Smith and Nichols, 2009), for visualization and secondary analyses.

### Statistical analysis

All statistical analyses were performed using R version 4.3.2. Continuous variables are presented as mean ± standard deviation or median with interquartile range, and categorical variables as number and percentage. Group differences were assessed using one-way analysis of variance or the Kruskal–Wallis test for continuous variables and the χ^2^ test or Fisher’s exact test for categorical variables. When the overall comparison was significant, pairwise *post-hoc* tests were performed with Holm adjustment. Serum sTREM2, WMH volume, and high-sensitivity C-reactive protein (hs-CRP) were natural-log transformed because of right-skewed distributions and then standardized before regression analyses.

Multivariable regression models were used to examine associations of serum sTREM2 with CSVD burden, MRI markers, and cognitive performance. Unless otherwise specified, models were adjusted for age, sex, years of education, body mass index, hypertension, diabetes, hyperlipidemia, and total intracranial volume. hs-CRP was additionally included in analyses of MRI markers to account for systemic inflammation. CSVD burden was analyzed as both a categorical variable and a continuous score. Among participants with CSVD, linear regression was used for WMH volume, Poisson or negative binomial regression was used for lacune and CMB counts according to the presence of overdispersion, and logistic regression was used for PVS > 10.

For diffusion MRI analyses, mean FA and MD values were extracted from significant TBSS clusters associated with serum sTREM2. Partial correlations between extracted diffusion metrics and cognitive measures were adjusted for age, sex, years of education, body mass index, hypertension, diabetes, and hyperlipidemia. Multiple comparisons were controlled using the Benjamini–Hochberg false discovery rate method.

To test whether the association between serum sTREM2 and global cognition differed according to CSVD burden among participants with CSVD, the standardized MoCA score was used as the primary cognitive outcome, with the standardized MMSE score examined as complementary measure of global cognition. For each outcome, standardized log-transformed serum sTREM2, burden group, and their interaction term were included as the main predictors. Models were adjusted for age, sex, years of education, body mass index, hypertension, diabetes, hyperlipidemia, and total intracranial volume. To provide inference robust to potential heteroscedasticity, 95% confidence intervals and *P*-values for the interaction terms were calculated using HC3 heteroscedasticity-consistent standard errors; these HC3-based *P*-values are reported for the interaction analyses in the main text and figures. Because MoCA and MMSE addressed the same hypothesis regarding global cognition, their interaction tests were treated as a single inferential family, and the HC3-based *P*-values were further adjusted using the Holm procedure (Supplementary Table 2). The robustness of the primary MoCA interaction was additionally evaluated using burden-stratified bootstrap resampling with 5,000 iterations and leave-one-out analysis (Supplementary Table 3). The same interaction model was additionally applied to a composite measure of executive function and processing speed as a secondary domain-specific analysis (Supplementary Table 4). WMH volume was not included because it is a component of the total CSVD burden score, and additional adjustment could introduce overadjustment.

In exploratory analyses restricted to participants with high-burden CSVD, we examined whether serum sTREM2 was associated with cognitive performance beyond established clinical and imaging factors. MoCA residuals were first derived from a model including age, sex, years of education, body mass index, hypertension, diabetes, hyperlipidemia, total intracranial volume, log-transformed WMH volume, and MD of the body of the corpus callosum. The association between standardized log-transformed serum sTREM2 and these MoCA residuals was then examined using linear regression, with 95% confidence intervals and *P*-values calculated using HC3 heteroscedasticity-consistent robust standard errors. We further constructed nested linear models to evaluate the incremental explanatory contribution of serum sTREM2 to MoCA variability. Standardized MoCA score was used as the dependent variable. Model 1 included demographic and vascular covariates together with total intracranial volume; Model 2 additionally included log-transformed WMH volume; Model 3 further included MD of the body of the corpus callosum; and Model 4 added standardized log-transformed serum sTREM2. All models were fitted in the same complete-case sample, and incremental model performance was assessed using R^2^, adjusted R^2^, change in R^2^, partial R^2^, AIC, and nested model comparisons. Given the limited sample size of the high-burden CSVD subgroup, additional sensitivity analyses were performed using Cook’s distance, leave-one-out refitting, Huber M-estimation, and nonparametric bootstrap resampling with 2,000 iterations. Bootstrap internal validation with 2,000 resamples was additionally used to estimate optimism-corrected R^2^ for Models 3 and 4 (Supplementary Table 5). HC3 heteroscedasticity-consistent robust standard errors were used where appropriate, and two-sided *P*-values < 0.05 were considered statistically significant.

## Results

### Participant characteristics

A total of 171 participants were included in the analysis, comprising 43 healthy controls, 83 participants with low-burden CSVD, and 45 participants with high-burden CSVD. Baseline characteristics are summarized in Table 1. Hypertension and hyperlipidemia differed significantly across groups, whereas sex, age, years of education, body mass index, diabetes, and total intracranial volume were comparable. Cognitive performance declined with increasing CSVD burden, accompanied by higher WMH volume and serum sTREM2 levels.

**TABLE 1:** Participant characteristics.

Variable	HC	L-CSVD	H-CSVD	*P*
Sex (male), *n* (%)	21 (48.84%)	50 (60.24%)	26 (57.78%)	0.466
Hypertension, *n* (%)	17 (39.53%)	54 (65.06%)	32 (71.11%)	0.005^ab^
Diabetes, *n* (%)	6 (13.95%)	9 (10.84%)	7 (15.56%)	0.727
Hyperlipidemia, *n* (%)	6 (13.95%)	27 (32.53%)	6 (13.33%)	0.013
Age, years	59.65 ± 7.52	62.31 ± 9.03	63.27 ± 9.07	0.127
Years of education	8.35 ± 3.79	9.13 ± 4.19	8.30 ± 3.97	0.425
BMI	24.17 ± 2.60	23.82 ± 3.16	24.28 ± 2.76	0.660
MMSE	27.35 ± 2.18	26.42 ± 2.90	24.42 ± 4.63	< 0.001^abc^
MoCA	23.77 ± 2.79	22.77 ± 3.95	20.40 ± 4.81	< 0.001^bc^
Immediate recall	15.77 ± 5.19	15.28 ± 4.41	13.13 ± 5.07	0.020
5-min delayed recall	5.37 ± 2.29	5.23 ± 2.45	4.04 ± 2.37	0.013^bc^
20-min delayed recall	4.84 ± 2.51	4.96 ± 2.47	3.71 ± 2.62	0.023^c^
Recognition	20.33 ± 2.87	20.22 ± 2.88	19.42 ± 3.61	0.298
CDR	0.22 ± 0.25	0.18 ± 0.25	0.36 ± 0.35	0.004^c^
Stroop(s)	38.24 ± 11.46	39.47 ± 13.87	43.90 ± 17.42	0.139
TMT-A(s)	74.91 ± 31.76	74.64 ± 39.77	92.11 ± 42.51	0.038
BNT	47.19 ± 7.85	48.37 ± 9.73	42.93 ± 12.14	0.014^c^
sTREM2, pg/mL	32.57 ± 20.84	34.73 ± 17.49	48.59 ± 29.02	< 0.001^bc^
hs-CRP, mg/L	2.95 ± 4.63	1.36 ± 1.48	2.94 ± 4.71	0.018
WMH volume, mL	2.52 ± 1.67	9.83 ± 11.72	26.60 ± 22.40	< 0.001^abc^
TIV, mL	1484.86 ± 116.53	1514.52 ± 134.75	1490.99 ± 89.84	0.344

Continuous variables are presented as mean ± SD and categorical variables as *n* (%). Overall *P*-values are from one-way ANOVA (or Kruskal–Wallis when assumptions were not met) for continuous variables and χ^2^ (or Fisher’s exact) tests for categorical variables. When the overall test was significant, pairwise *post-hoc* comparisons were performed with Holm adjustment. Superscripts indicate significant pairwise differences after adjustment: ^a^HC vs. L-CSVD; ^b^HC vs. H-CSVD; ^c^L-CSVD vs. H-CSVD. HC, healthy controls; CSVD, cerebral small-vessel disease; L-CSVD, low CSVD burden (total score 1–2); H-CSVD, high CSVD burden (total score 3–4); BMI, body mass index; MMSE, Mini-Mental State Examination; MoCA, Montreal Cognitive Assessment; CDR, Clinical Dementia Rating; BNT, Boston Naming Test; AVLT, Auditory Verbal Learning Test; sTREM2, soluble triggering receptor expressed on myeloid cells 2; hs-CRP, high-sensitivity C-reactive protein; WMH volume, white-matter hyperintensity volume; TIV, total intracranial volume; TMT-A, Trail Making Test Part A.

### Serum sTREM2 increases with CSVD burden

Serum sTREM2 levels differed significantly across the CSVD burden spectrum in multivariable-adjusted models (adjusted overall group effect, *P* = 0.001; Figure 1A). Holm-corrected pairwise comparisons showed that serum sTREM2 was significantly higher in the high-burden CSVD group than in both healthy controls (*P* = 0.001) and low-burden CSVD participants (*P* = 0.007), whereas no significant difference was observed between healthy controls and low-burden CSVD participants (*P* = 0.267).

**FIGURE 1:**
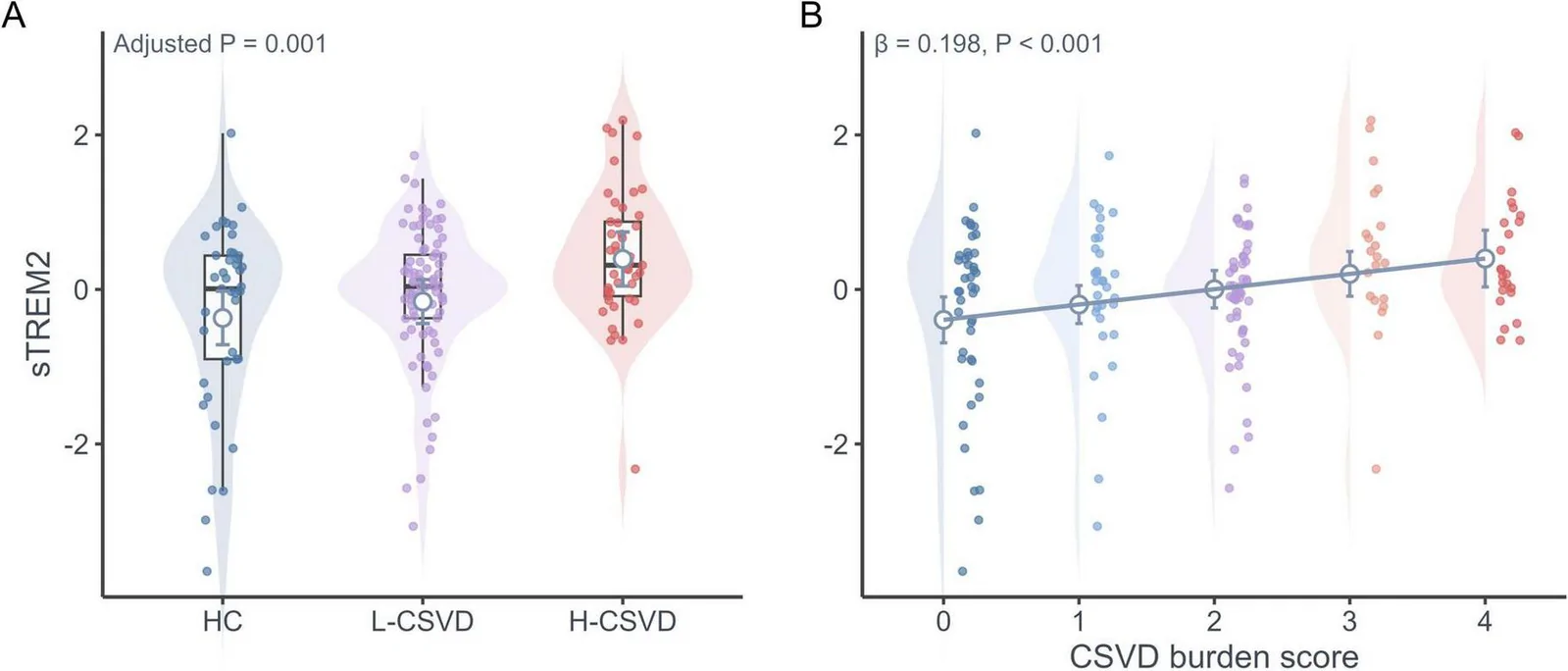
Serum sTREM2 increases with CSVD burden. **(A)** Distribution of serum sTREM2 across healthy controls (HC), low-burden CSVD (L-CSVD; CSVD score 1–2), and high-burden CSVD (H-CSVD; CSVD score 3–4). Violin plots and jittered points show the distribution of individual values, boxplots indicate the median and interquartile range, and open circles with error bars represent covariate-adjusted marginal means and 95% confidence intervals. **(B)** Adjusted linear trend of serum sTREM2 across total CSVD burden scores from 0 to 4. Individual points and half-violin plots show the observed distribution, whereas open circles, error bars, and the connecting line represent adjusted marginal means and 95% confidence intervals estimated from the multivariable model. Statistical analyses were performed using z-standardized log-transformed serum sTREM2 and were adjusted for age, sex, education, body mass index, hypertension, diabetes, hyperlipidemia, and total intracranial volume.

When the total CSVD burden score was modeled as a continuous variable, higher CSVD burden was associated with higher serum sTREM2 (β = 0.198, SE = 0.058, *P* < 0.001; Figure 1B), consistent with a graded burden-dependent increase in serum sTREM2.

### Serum sTREM2 is associated with WMH burden but not focal CSVD imaging markers

We next examined whether serum sTREM2 was associated with individual MRI markers of CSVD. In models additionally adjusted for hs-CRP, higher serum sTREM2 was significantly associated with greater WMH volume (β = 0.208, 95% CI, 0.028 to 0.388, *P* = 0.026; Figure 2A). In contrast, serum sTREM2 was not significantly associated with lacunes, CMBs, or PVS > 10.

**FIGURE 2:**
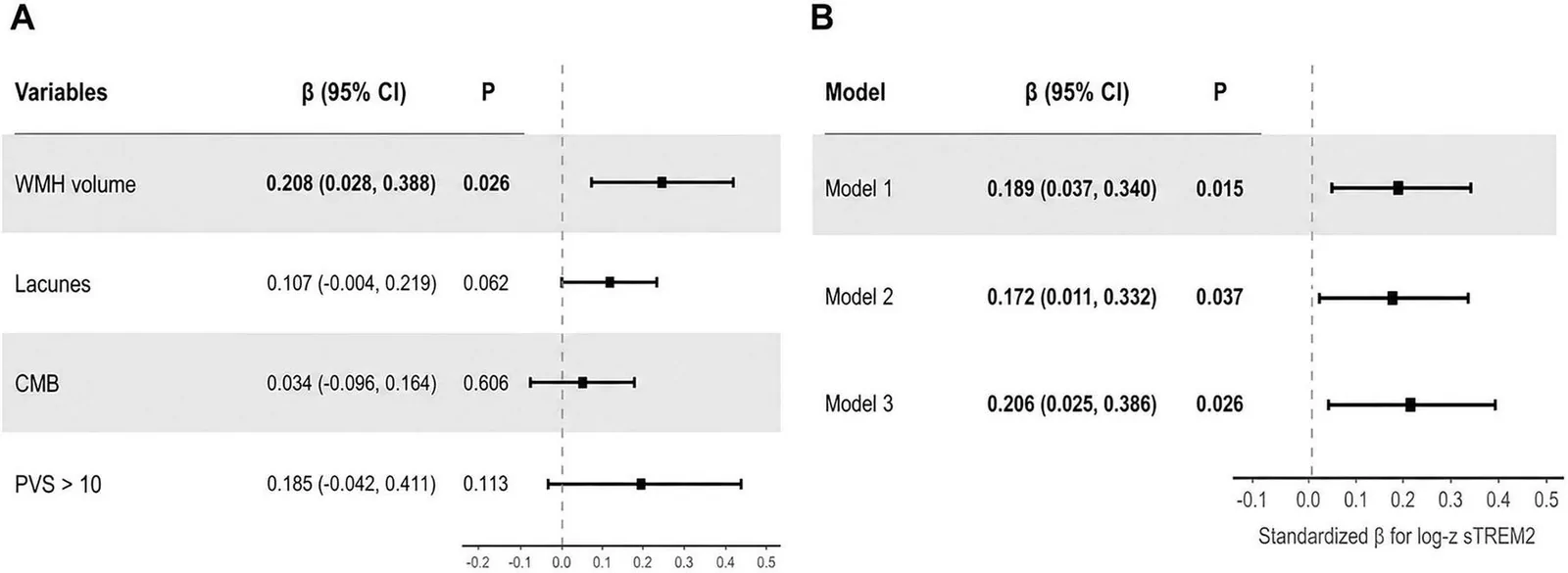
Serum sTREM2 is associated with WMH burden but not focal CSVD imaging markers. **(A)** Associations of serum sTREM2 with individual CSVD imaging markers, including WMH volume, lacune count, cerebral microbleed count, and enlarged perivascular spaces in the basal ganglia (PVS > 10). Linear, count, or logistic regression models were used according to outcome type. **(B)** Sequential multivariable models for the association between serum sTREM2 and WMH volume. Model 1 was adjusted for age, sex, education, body mass index, and total intracranial volume. Model 2 was additionally adjusted for hypertension, diabetes, and hyperlipidemia. Model 3 was further adjusted for hs-CRP. Squares indicate standardized β estimates; horizontal lines indicate 95% confidence intervals.

Sequential multivariable models further supported the association between serum sTREM2 and WMH volume. This association remained significant after adjustment for demographic variables (β = 0.189, 95% CI, 0.037 to 0.340, *P* = 0.015), further adjustment for vascular risk factors (β = 0.172, 95% CI, 0.011 to 0.332, *P* = 0.037), and additional adjustment for hs-CRP (β = 0.206, 95% CI, 0.025 to 0.386, *P* = 0.026; Figure 2B).

### Higher serum sTREM2 is associated with distributed MD abnormalities

Voxel-wise TBSS analysis was performed to localize white matter microstructural abnormalities associated with serum sTREM2. Higher serum sTREM2 was associated with increased mean diffusivity (MD) across multiple white matter tracts, including the splenium of the corpus callosum (SCC), left posterior thalamic radiation (PTR; including the optic radiation, OR), body of the corpus callosum (BCC), right superior longitudinal fasciculus (SLF), right superior corona radiata (SCR), bilateral external capsule (EC), and right cingulum (cingulate gyrus, CGC). By contrast, the association with fractional anisotropy (FA) was spatially restricted to lower FA in the right anterior corona radiata (ACR; Figure 3). This pattern is consistent with a closer relationship between serum sTREM2 and diffuse MD abnormalities than with widespread FA reductions.

**FIGURE 3:**
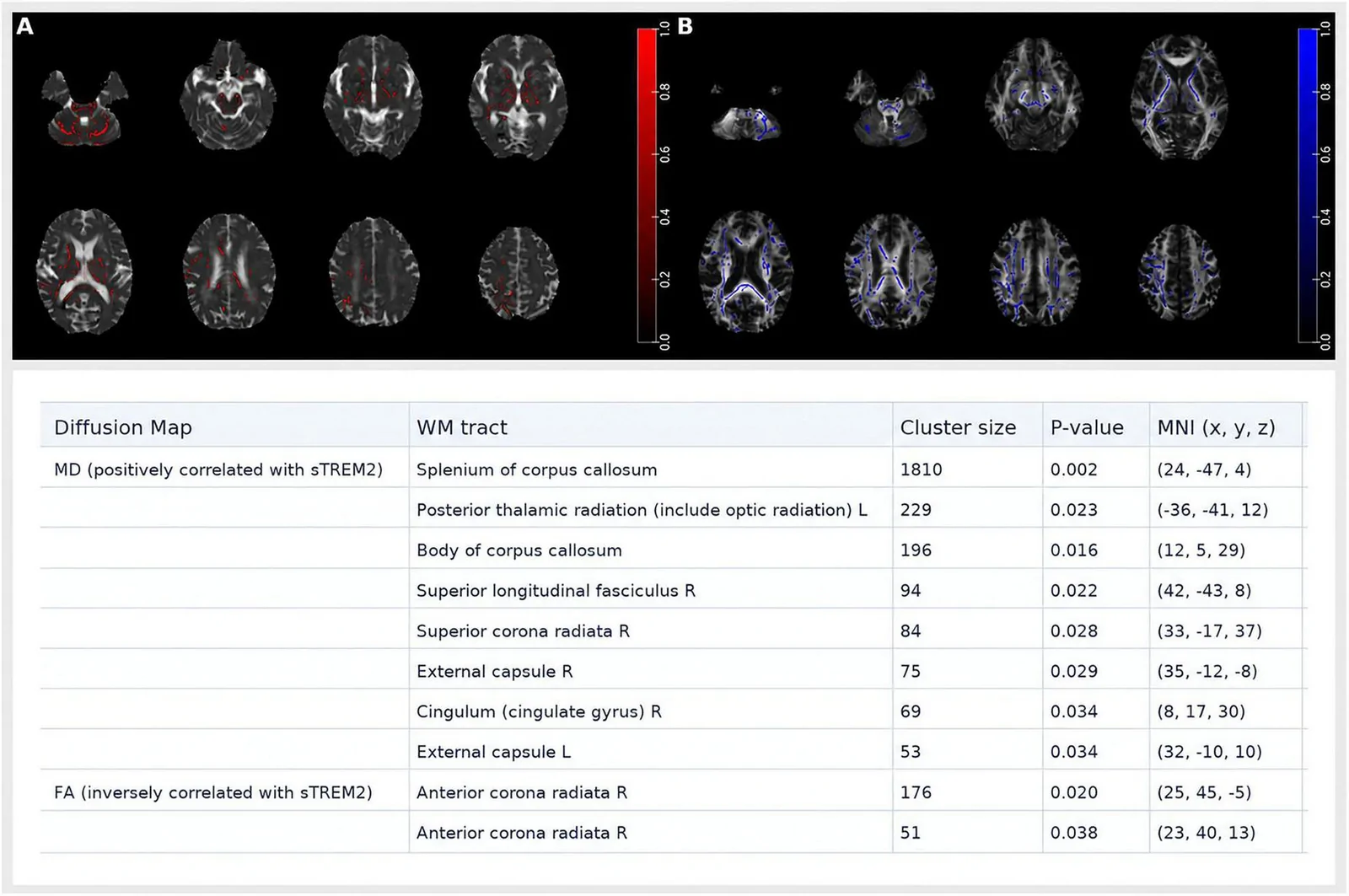
Higher serum sTREM2 is associated with distributed MD abnormalities. Permutation testing (5,000 permutations; threshold-free cluster enhancement, TFCE; family-wise error–corrected *p* < 0.05) revealed widespread positive associations of sTREM2 with mean diffusivity (MD), predominantly involving the corpus callosum, posterior thalamic radiation, superior longitudinal fasciculus, superior corona radiata, external capsule, and cingulum. Fractional anisotropy (FA) showed a negative association with sTREM2 in the right anterior corona radiata. Models were adjusted for age, sex, and years of education. **(A)** displays brain regions where mean diffusivity (MD) is positively correlated with serum sTREM2 (shown in red), whereas **(B)** illustrates regions where fractional anisotropy (FA) is negatively correlated with sTREM2 (shown in blue).

### Cognitive relevance of sTREM2-associated diffusion abnormalities

After adjustment for demographic and vascular risk factors, MD metrics showed broader and more consistent cognitive associations than FA metrics (Figure 4). Among the extracted regions, MD-BCC showed the most prominent associations, including lower MMSE, lower MoCA, poorer delayed recall, lower naming performance, and longer TMT-A completion time after FDR correction. MD-SCC and MD-PTR were also associated with several cognitive measures, whereas FA-ACR showed limited cognitive associations.

**FIGURE 4:**
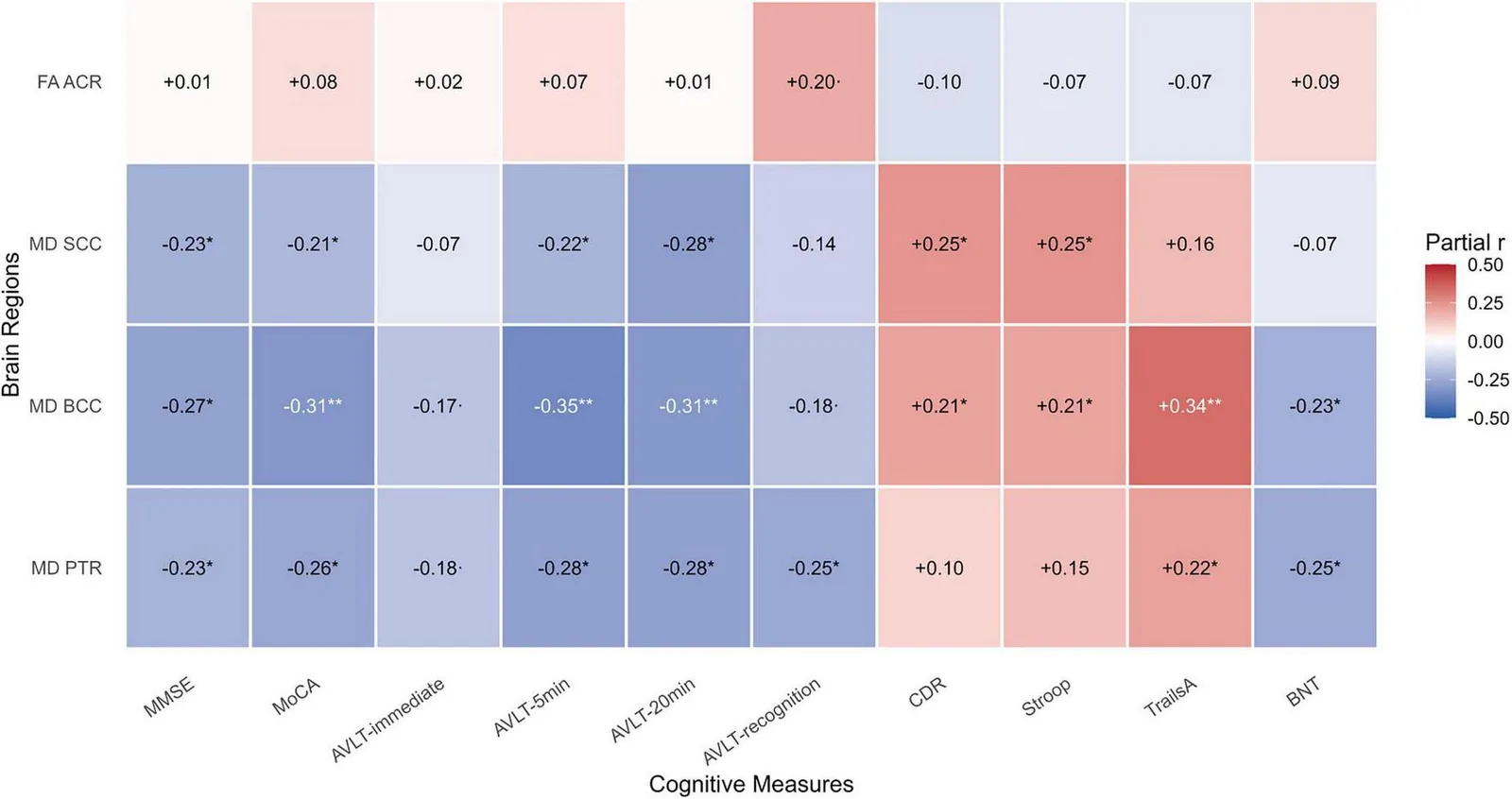
Cognitive relevance of sTREM2-associated diffusion abnormalities. The heatmap shows partial correlation coefficients between selected DTI-derived white matter metrics and cognitive measures after adjustment for sex, age, education, BMI, hypertension, diabetes, and hyperlipidemia. Blue indicates negative correlations, whereas red indicates positive correlations. Values within each cell represent partial correlation coefficients. Statistical significance was corrected using the false discovery rate method. For CDR, Stroop, and TMT-A, higher values indicate worse performance; therefore, the clinical direction of correlations should be interpreted according to each test. ^⋅^FDR-adjusted *P* < 0.10; *FDR-adjusted P < 0.05; **FDR-adjusted *P* < 0.01; ***FDR-adjusted *P* < 0.001. ACR, anterior corona radiata; SCC, splenium of the corpus callosum; BCC, body of the corpus callosum; PTR, posterior thalamic radiation; FA, fractional anisotropy; MD, mean diffusivity; AVLT, Auditory Verbal Learning Test; CDR, Clinical Dementia Rating; BNT, Boston Naming Test.

### The association between serum sTREM2 and global cognition differs by CSVD burden

The association between serum sTREM2 and global cognition differed according to CSVD burden. For MoCA, a significant interaction was observed between standardized log-transformed serum sTREM2 and CSVD burden (β = 0.462, 95% CI, 0.054 to 0.869; HC3 *P* = 0.027; Holm-adjusted *P* = 0.027; Figure 5A). The corresponding interaction for MMSE also remained significant after Holm correction (β = 0.531, 95% CI, 0.124 to 0.938; HC3 *P* = 0.011; Holm-adjusted *P* = 0.022; Supplementary Table 2). The primary MoCA interaction was further supported by complementary robustness analyses. Burden-stratified bootstrap resampling yielded a median interaction coefficient of 0.455 (95% bootstrap CI, 0.135 to 0.873), while leave-one-out refitting produced consistently positive estimates (mean β = 0.462; range, 0.382 to 0.583; Supplementary Table 3). In the secondary exploratory domain-specific analysis, no significant interaction between serum sTREM2 and CSVD burden was observed for the composite measure of executive function and processing speed (β = 0.105, 95% CI, −0.381 to 0.591; *P* = 0.669; Supplementary Table 4). Burden-stratified estimates showed no significant association between serum sTREM2 and MoCA performance in the low-burden CSVD group (β = −0.077, 95% CI, −0.303 to 0.150, *P* = 0.504). In contrast, higher serum sTREM2 was associated with higher MoCA scores in the high-burden CSVD group (β = 0.385, 95% CI, 0.051 to 0.718, *P* = 0.024). These findings suggest that the association between serum sTREM2 and global cognition differed by CSVD burden and was mainly observed among participants with high overall disease burden.

**FIGURE 5:**
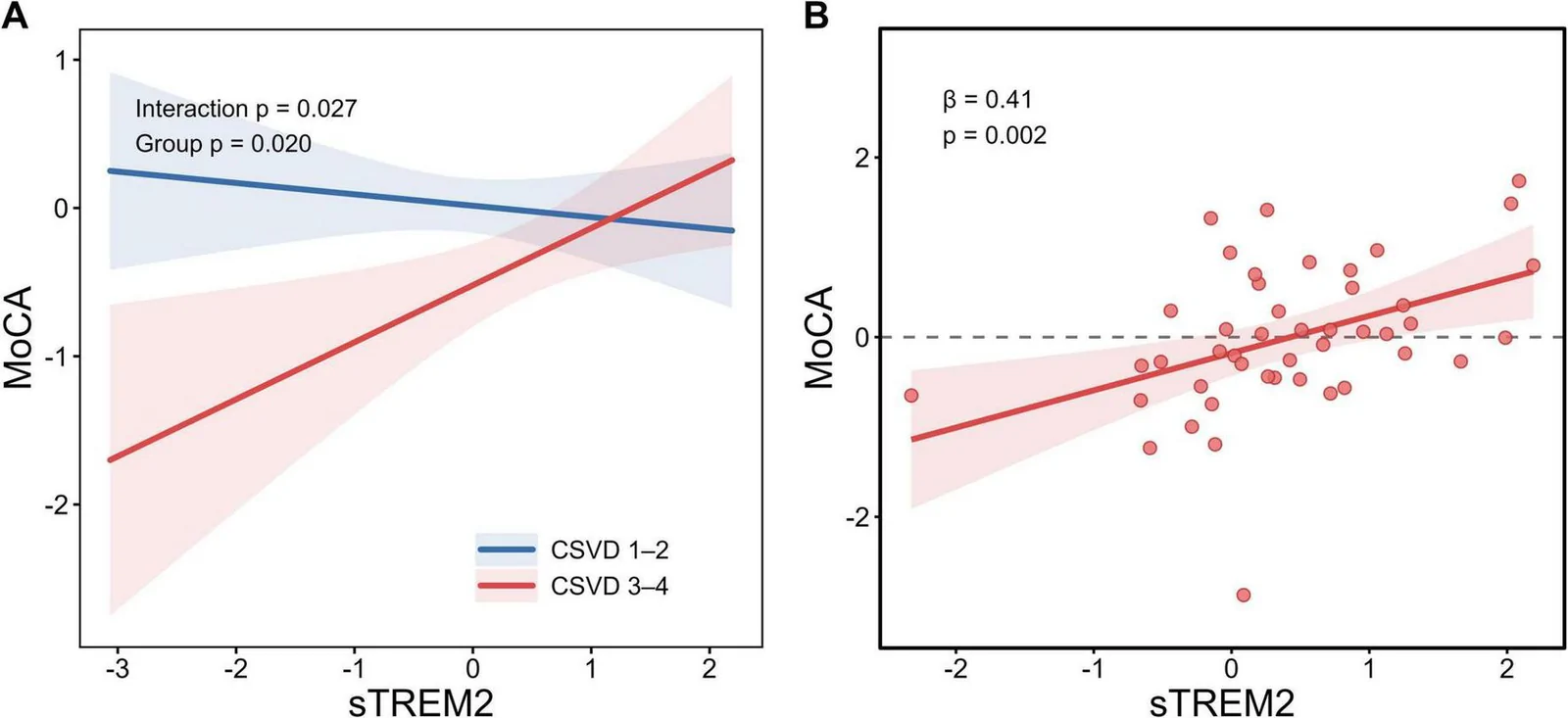
The association between serum sTREM2 and global cognition differs by CSVD burden. **(A)** Adjusted interaction plot showing the association between serum sTREM2 and MoCA performance in low-burden CSVD (CSVD score 1–2) and high-burden CSVD (CSVD score 3–4). Serum sTREM2 was natural-log transformed and standardized, and MoCA scores were standardized before modelling. Lines represent model-based fitted values, and shaded areas indicate 95% confidence intervals. The interaction model was adjusted for age, sex, education, body mass index, hypertension, diabetes, hyperlipidemia, and total intracranial volume. WMH volume was not included because it is a component of the total CSVD burden score. Confidence intervals and *P*-values for the interaction term were calculated using HC3 heteroscedasticity-consistent robust standard errors. **(B)** Scatter plot showing the association between serum sTREM2 and MoCA residuals among participants with high-burden CSVD. MoCA residuals were derived after adjustment for age, sex, education, body mass index, hypertension, diabetes, hyperlipidemia, total intracranial volume, log-transformed WMH volume, and MD of the body of the corpus callosum. The dashed horizontal line indicates a residual value of zero; positive residuals indicate better-than-expected MoCA performance relative to the included clinical and imaging variables. The association between standardized log-transformed serum sTREM2 and MoCA residuals was evaluated using linear regression, with confidence intervals and *P*-values calculated using HC3 heteroscedasticity-consistent robust standard errors.

To examine whether serum sTREM2 was associated with residual cognitive variation after accounting for measured white matter injury, MoCA residuals were derived after adjustment for demographic factors, vascular risk factors, total intracranial volume, log-transformed WMH volume, and MD of the body of the corpus callosum. In this subgroup, higher serum sTREM2 was associated with higher MoCA residuals (β = 0.415, 95% CI, 0.161 to 0.669; HC3 *P* = 0.002; Figure 5B). To further quantify the incremental explanatory information provided by serum sTREM2, nested models were constructed among participants with high-burden CSVD (Table 2). After clinical covariates, WMH volume, and MD-BCC were included, adding serum sTREM2 increased the model R^2^ from 0.496 to 0.602 (ΔR^2^ = 0.105; partial R^2^ = 0.209) and improved model fit (ΔAIC = −8.54; incremental *F* = 8.71, *P* = 0.006). In the final model, serum sTREM2 showed a positive association with MoCA performance (β = 0.469, 95% CI, 0.146 to 0.792, *P* = 0.006). These findings were consistent across complementary sensitivity analyses (Supplementary Table 5). Although bootstrap internal validation indicated optimism in the apparent R^2^ estimates, the model including serum sTREM2 retained a higher optimism-corrected R^2^ than Model 3 (0.304 vs. 0.141).

**TABLE 2:** Incremental explanatory value of serum sTREM2 for MoCA performance in high-burden CSVD.

Model	R^2^	Adjusted R^2^	AIC	ΔR^2^	Partial R^2^	ΔAIC	*F*	*P*-value
Model 1	0.395	0.260	137.82	–	–	–	–	–
Model 2	0.431	0.284	137.04	0.036	0.060	−0.78	2.23	0.144
Model 3	0.496	0.348	133.53	0.066	0.115	−3.51	4.43	0.043
Model 4	0.602	0.469	124.99	0.105	0.209	−8.54	8.71	0.006

All models were fitted in the same complete-case sample of participants with high-burden CSVD (*n* = 45). Model 1 included age, sex, years of education, body mass index, hypertension, diabetes, hyperlipidemia, and total intracranial volume. Model 2 additionally included log-transformed white matter hyperintensity volume. Model 3 additionally included mean diffusivity of the body of the corpus callosum. Model 4 additionally included log-transformed serum sTREM2. Values for ΔR^2^, partial R^2^, ΔAIC, F, and *P*-value indicate the incremental improvement relative to the preceding model. AIC, Akaike information criterion; CSVD, cerebral small vessel disease; MD-BCC, mean diffusivity of the body of the corpus callosum; MoCA, Montreal Cognitive Assessment; sTREM2, soluble triggering receptor expressed on myeloid cells 2; TIV, total intracranial volume; WMH, white matter hyperintensity.

## Discussion

In this MRI-defined cerebral small vessel disease cohort, serum sTREM2 was associated with WMH volume and MD-based white matter abnormalities, whereas no significant associations were observed with lacunes, CMBs, or PVS. Its relationship with global cognition was not uniform across the disease spectrum, but was most evident in high-burden disease. These findings suggest that serum sTREM2 may provide additional information for characterizing white matter–dominant imaging burden and cognitive heterogeneity in cerebral small vessel disease.

The association between serum sTREM2 and WMH volume further extends previous evidence linking circulating sTREM2 to cerebrovascular white matter injury, including the association between plasma sTREM2 and white matter lesions independent of amyloid and tau pathology (Rodriguez-Vieitez and Ashton, 2021; Tsai et al., 2021). In CSVD, WMH abnormalities capture the chronic and diffuse dimensions of white matter damage, which may arise from small vessel dysfunction, hypoperfusion, blood brain barrier disruption, impaired clearance of brain interstitial fluid, and injury to myelin and axonal units(Shi and Wardlaw, 2016; Wardlaw et al., 2013). These processes may increase the demand for lipid handling, clearance of myelin debris, and tissue remodeling, processes in which TREM2-related myeloid pathways have been implicated (Cantoni et al., 2015; Farkas et al., 2004; Schlepckow et al., 2017; Shibata et al., 2004). In contrast, lacunes, CMBs, and PVS may reflect more heterogeneous or compartment-specific aspects of small vessel disease. Therefore, the observed associations of serum sTREM2 with WMH volume and MD abnormalities, together with the absence of significant associations with focal CSVD markers, are consistent with a potential relationship between circulating sTREM2 and diffuse white matter injury in CSVD.

The diffusion MRI findings extend the WMH results by suggesting that serum sTREM2 is related not only to macroscopic WMH burden, but also to more diffuse white matter microstructural abnormalities. In the TBSS analysis, higher serum sTREM2 was associated with distributed increases in MD involving major white matter tracts, whereas FA reductions were spatially limited. This MD-predominant pattern is relevant in CSVD because increased MD may capture diffuse alterations in the white matter microenvironment, such as increased water mobility, extracellular-space expansion, and reduced microstructural barriers, whereas FA is more dependent on the directional coherence of fiber organization and may be less sensitive to tissue changes in regions with complex or partially disrupted fiber architecture (Amlien et al., 2013; Horsfield and Jones, 2002; Kim et al., 2023). The clinical relevance of this pattern was supported by the cognitive correlation analyses. MD measures showed broader associations with cognitive performance than FA measures (Maniega et al., 2015; Wardlaw et al., 2015), and MD in the body of the corpus callosum showed the most consistent associations with global cognition, delayed memory, processing speed, and executive function. The corpus callosum is a major commissural pathway involved in interhemispheric communication and distributed cognitive processing, and previous diffusion MRI studies have linked callosal and other white matter microstructural abnormalities to cognitive impairment in CSVD and aging-related cognitive disorders (Dobrynina et al., 2024; Qiao et al., 2021; Wang et al., 2024; Yeske et al., 2021). Together with the WMH findings, these results suggest that the observed associations of serum sTREM2 were concentrated in white matter–related imaging measures that have cognitive relevance. However, the present cross-sectional data cannot determine whether serum sTREM2 reflects a compensatory tissue response, a marker of injury burden, or both.

These findings raise a broader interpretive question: why would a circulating marker that increases with CSVD burden and white matter abnormalities show a positive cognitive association mainly in high-burden CSVD? One possible explanation is that the meaning of serum sTREM2 may differ when considered across the disease spectrum versus within individuals who already have a high cumulative CSVD burden. Across the full CSVD spectrum, higher serum sTREM2 may primarily accompany greater white matter–dominant disease burden, as reflected by its associations with total CSVD burden, WMH volume, and MD abnormalities. Within the high-burden group, however, differences in serum sTREM2 may capture additional biological heterogeneity that is not fully represented by conventional measures of white matter injury. Consistent with this possibility, serum sTREM2 was positively associated with MoCA residuals after accounting for demographic and vascular factors, WMH volume, and MD of the body of the corpus callosum. Together, these findings raise the possibility that, in the setting of substantial CSVD-related structural injury, variation in circulating sTREM2 may capture aspects of TREM2-related myeloid responses that are relevant to cognitive heterogeneity.

A similar context-dependent interpretation has been proposed in Alzheimer disease, where the clinical meaning of sTREM2 appears to depend on the underlying pathological burden. Without accounting for amyloid or tau pathology, higher sTREM2 may mainly reflect more active disease (Piccio et al., 2016; Suárez-Calvet et al., 2016); at a given level of pathology, however, higher sTREM2 has been associated with slower cognitive or clinical decline (Biel et al., 2023; Ewers et al., 2019). By analogy, serum sTREM2 in CSVD may have different implications depending on the extent of white matter disease. Across the burden spectrum, it may track diffuse white matter injury; within high-burden CSVD, it may instead index variability in TREM2-related myeloid responses associated with established tissue injury, potentially involving lipid handling, myelin-debris clearance, phagocytosis, and tissue remodeling (Biel et al., 2023; McCray et al., 2024). This remains a hypothesis, particularly because serum sTREM2 may arise from both central and peripheral myeloid compartments and cannot establish protective effects in a cross-sectional study. Nevertheless, this framework may explain why the cognitive relevance of serum sTREM2 emerged primarily in high-burden CSVD, whereas at lower burden other determinants of cognitive performance, such as cognitive reserve, lesion location, vascular risk factors, and systemic inflammation, may be more influential (Franceschi et al., 2018; Poliani et al., 2015).

Several limitations should be acknowledged. First, the cross-sectional design precludes inference about temporal sequence or causality; therefore, the present findings should not be interpreted as evidence that serum sTREM2 causes white matter injury or directly protects cognition. Longitudinal studies with repeated biomarker, MRI, and cognitive assessments are needed to determine whether serum sTREM2 predicts WMH progression, diffusion abnormalities, or cognitive decline. Second, serum sTREM2 is not a direct measure of central TREM2-related microglial activity and may also be influenced by peripheral myeloid activity, systemic inflammation, or vascular comorbidities. Nevertheless, serum biomarkers are clinically accessible, and the observed associations remained evident after adjustment for demographic, vascular, and inflammatory factors. Third, the high-burden CSVD subgroup was relatively small, which limits the precision of the burden-dependent interaction and burden-stratified estimates. Although the primary MoCA interaction remained directionally stable in burden-stratified bootstrap and leave-one-out analyses, these analyses provide only internal evidence of robustness and cannot substitute for replication in an independent cohort. The additional analyses restricted to the high-burden subgroup, including the residualized MoCA and nested-model analyses, should be interpreted even more cautiously because they were conducted in the same limited sample and may be susceptible to overfitting and optimistic estimates of model performance. Bootstrap internal validation reduced, but did not eliminate, this concern. Accordingly, these subgroup findings should be considered exploratory and require confirmation in larger, independent cohorts. Finally, DTI metrics are not histopathologically specific, limiting interpretation of the cellular and tissue processes underlying the observed white matter microstructural abnormalities.

## Conclusion

In conclusion, serum sTREM2 was associated with total cerebral small vessel disease burden, white matter hyperintensity volume, and diffusion magnetic resonance imaging–derived mean diffusivity abnormalities. Its relationship with cognition was most evident in high-burden disease, suggesting that serum sTREM2 may provide additional information for understanding cognitive heterogeneity beyond conventional magnetic resonance imaging lesion burden. Longitudinal studies are needed to clarify its prognostic value and clinical relevance.

## Data Availability

The raw data supporting the conclusions of this article will be made available by the authors, without undue reservation.
