# Supplementary material for: Burden-dependent associations of serum sTREM2 with white matter injury and cognitive heterogeneity in cerebral small vessel disease
Source: Front Aging Neurosci. 2026 Sep 21;18:1937413. doi: 10.3389/fnagi.2026.1937413 (PMC13635210; doi:10.3389/fnagi.2026.1937413)
Supplement: Supplementary file 1 [file Data_Sheet_1.docx]

**Supplementary Table 1. Inter-rater reliability of CSVD imaging assessments**

| **Imaging marker** | **N** | **Reliability statistic** | **Estimate** | **95% CI** |
| --- | --- | --- | --- | --- |
| WMH volume | 128 | ICC(A,1) | 0.975 | 0.957–0.987 |
| Lacunes | 128 | Cohen’s κ | 0.867 | 0.771–0.950 |
| CMBs | 128 | Cohen’s κ | 0.875 | 0.781–0.953 |
| PVS >10 | 128 | Cohen’s κ | 0.899 | 0.810–0.968 |

**Note:** Inter-rater reliability was evaluated among the 128 participants with CSVD.WMH volume was assessed using a two-way mixed-effects intraclass correlation coefficient for absolute agreement based on single measurements [ICC(A,1)]. Inter-rater agreement for lacunes, cerebral microbleeds (CMBs), and perivascular spaces (PVS; >10) was assessed using Cohen’s κ. Values are reported with 95% confidence intervals (CIs).

**Supplementary Table 2. Global cognitive interaction analyses with Holm correction**

| **Outcome** | **N** | **Interaction β** | **95% CI** | **HC3 P** | **Holm-adjusted P** |
| --- | --- | --- | --- | --- | --- |
| MoCA | 128 | **0.462** | 0.054 to 0.869 | 0.027 | **0.027** |
| MMSE | 128 | **0.531** | 0.124 to 0.938 | 0.011 | **0.022** |

**Note:** Interaction β represents the coefficient for z-standardized ln(sTREM2) by CSVD burden group (CSVD score 1–2 vs 3–4). Models were adjusted for age, sex, education, body mass index, hypertension, diabetes, hyperlipidemia, and total intracranial volume. Confidence intervals and unadjusted P values were calculated using HC3 heteroscedasticity-consistent robust standard errors. Holm correction was subsequently applied to the two HC3 P values for the MoCA and MMSE interaction tests.

**Supplementary Table 3. Resampling and influence analyses of the interaction coefficient**

| **Analysis** | **No. of iterations** | **Interaction β** | **95% CI / range** |
| --- | --- | --- | --- |
| Burden-stratified bootstrap | 5,000 | 0.455* | 0.135 to 0.873 |
| Leave-one-out analysis | 128 | 0.462† | 0.382 to 0.583‡ |

**Note:** *Median bootstrap interaction coefficient; the interval represents the percentile-based 95% bootstrap confidence interval. †Mean interaction coefficient across leave-one-out refits. ‡Minimum-to-maximum interaction coefficient across the 128 leave-one-out refits; this range is not a confidence interval.

**Supplementary Table 4. Interaction analysis of executive function and processing speed**

| **Outcome** | **N** | **Interaction β** | **95% CI** | **P value** |
| --- | --- | --- | --- | --- |
| executive function and processing speed composite | 128 | 0.105 | −0.381 to 0.591 | 0.669 |

**Note**: The executive function and processing speed composite was calculated as the mean of reverse-coded z-standardized natural-log-transformed Stroop-C and Trail Making Test Part A (TMT-A) completion times, such that higher values indicated better performance. The interaction coefficient represents the standardized ln(sTREM2) × CSVD burden-group term (low burden, score 1–2; high burden, score 3–4). The model was adjusted for age, sex, years of education, body mass index, hypertension, diabetes, hyperlipidemia, and total intracranial volume. HC3 heteroscedasticity-consistent robust standard errors were used for inference.

**Supplementary Table 5. Robustness and internal validation of the serum sTREM2–MoCA association in high-burden CSVD**

**A. Robustness of the sTREM2 coefficient**

| **Analysis** | **sTREM2 β (95% CI)** | **P value** |
| --- | --- | --- |
| Primary OLS model | 0.469 (0.146–0.792) | 0.006 |
| Leave-one-out analysis | 0.383–0.532 | 0.002–0.028 |
| Huber M-estimation | 0.440 (0.193–0.687) | — |
| Nonparametric bootstrap, 2,000 resamples | 0.444 (0.130–0.797) | — |

**B. Bootstrap internal validation of model performance**

| **Model** | **Apparent R²** | **Mean bootstrap optimism** | **Optimism-corrected R²** |
| --- | --- | --- | --- |
| Model 3: | 0.496 | 0.356 | 0.141 |
| Model 4 | 0.602 | 0.297 | 0.304 |

**Note:** All sensitivity analyses used the same high-burden CSVD complete-case sample (n = 45) and the same Model 4 specification as the primary analysis. Leave-one-out values represent the range across 45 refitted models. Robust regression was performed using Huber M-estimation. The nonparametric bootstrap estimate represents the mean sTREM2 coefficient across 2,000 successful resamples, and the corresponding 95% confidence interval was obtained using the percentile method. For bootstrap internal validation, Models 3 and 4 were evaluated separately using 2,000 valid resamples. In each resample, optimism was calculated as the difference between R² in the bootstrap sample and R² obtained when the bootstrap-fitted model was applied to the original sample; mean optimism was then subtracted from the apparent R² to obtain the optimism-corrected R². Resamples in which a categorical covariate contained only one observed level or the model failed to fit were discarded from the internal-validation procedure, and resampling continued until 2,000 valid iterations were obtained. Influence diagnostics identified three observations above the conventional Cook’s distance threshold (maximum D = 0.566; 4/n = 0.089); no observations were excluded. Values are rounded for presentation; changes in R² were calculated using the unrounded model estimates and may therefore differ slightly from differences calculated directly from the displayed values.
