# Supplementary material for: Burden-dependent associations of serum sTREM2 with white matter injury and cognitive heterogeneity in cerebral small vessel disease
Source: Front Aging Neurosci. 2026 Sep 21;18:1937413. doi: 10.3389/fnagi.2026.1937413 (PMC13635210; doi:10.3389/fnagi.2026.1937413)
Supplement: Supplementary file 2 [file Supplementary_file_1.docx]

**Supplementary Methods**

**Detailed assessment of cerebral small vessel disease markers**

White matter hyperintensities (WMH) were evaluated on T2-weighted and fluid-attenuated inversion recovery images using the Fazekas scale. Periventricular WMH and deep WMH were each graded from 0 to 3, with higher scores indicating greater lesion severity. WMH presence was defined as periventricular or deep WMH grade ≥1. Moderate-to-severe WMH was defined as periventricular WMH grade 3 and/or deep WMH grade 2–3. When an overall WMH grade was required, the higher grade of periventricular and deep WMH was used.

Lacunes were defined as round or ovoid cerebrospinal fluid-like cavities measuring 3–15 mm in diameter, located in the basal ganglia, thalamus, or deep white matter, with low signal intensity on T1-weighted images, high signal intensity on T2-weighted images, and a surrounding hyperintense rim on fluid-attenuated inversion recovery images.

Cerebral microbleeds (CMBs) were defined as small, rounded or ovoid hypointense lesions measuring <10 mm in diameter on susceptibility-weighted imaging or T2*-weighted gradient-echo images, according to established neuroimaging criteria.

Enlarged perivascular spaces were defined as punctate or linear structures with cerebrospinal fluid-like signal intensity on T2-weighted images and a diameter <3 mm. Perivascular spaces in the basal ganglia were rated using a 5-point scale according to lesion number: 0 = none, 1 = 1–10, 2 = 11–20, 3 = 21–40, and 4 = >40 .
